# Space use and social association in a gregarious ungulate: Testing the conspecific attraction and resource dispersion hypotheses

**DOI:** 10.1002/ece3.5071

**Published:** 2019-04-16

**Authors:** Mélissa Peignier, Quinn M. R. Webber, Erin L. Koen, Michel P. Laforge, Alec L. Robitaille, Eric Vander Wal

**Affiliations:** ^1^ Department of Biology Memorial University of Newfoundland St. John's Newfoundland Canada; ^2^ Cognitive and Behavioural Ecology Interdisciplinary Program Memorial University of Newfoundland St. John's Newfoundland Canada

**Keywords:** caribou, conspecific attraction hypothesis, home range overlap, resource dispersion hypothesis, social network analysis, spatial network

## Abstract

Animals use a variety of proximate cues to assess habitat quality when resources vary spatiotemporally. Two nonmutually exclusive strategies to assess habitat quality involve either direct assessment of landscape features or observation of social cues from conspecifics as a form of information transfer about forage resources. The conspecific attraction hypothesis proposes that individual space use is dependent on the distribution of conspecifics rather than the location of resource patches, whereas the resource dispersion hypothesis proposes that individual space use and social association are driven by the abundance and distribution of resources. We tested the conspecific attraction and the resource dispersion hypotheses as two nonmutually exclusive hypotheses explaining social association and of adult female caribou (*Rangifer tarandus*). We used location data from GPS collars to estimate interannual site fidelity and networks representing home range overlap and social associations among individual caribou. We found that home range overlap and social associations were correlated with resource distribution in summer and conspecific attraction in winter. In summer, when resources were distributed relatively homogeneously, interannual site fidelity was high and home range overlap and social associations were low. Conversely, in winter when resources were distributed relatively heterogeneously, interannual site fidelity was low and home range overlap and social associations were high. As access to resources changes across seasons, caribou appear to alter social behavior and space use. In summer, caribou may use cues associated with the distribution of forage, and in winter caribou may use cues from conspecifics to access forage. Our results have broad implications for our understanding of caribou socioecology, suggesting that caribou use season‐specific strategies to locate forage. Caribou populations continue to decline globally, and our finding that conspecific attraction is likely related to access to forage suggests that further fragmentation of caribou habitat could limit social association among caribou, particularly in winter when access to resources may be limited.

## 1 INTRODUCTION

Animals use a variety of proximate cues that might indicate habitat quality (Fletcher, 2006). One strategy is to use information acquired from the social environment as a cue for habitat quality (Merkle, Sigaud, & Fortin, 2015; Stamps, 1988). For example, some species use the reproductive success of conspecifics as a form of public information to choose their own breeding sites (Doligez, Danchin, & Clobert, 2002). Other species use the presence of foraging conspecifics as an index of patch quality, decreasing the costs of searching for forage (Kawaguchi, Ohashi, & Toquenaga, 2006). A second strategy is to use landscape features as a proxy for habitat quality. Some individuals, for example, select habitats to maximize camouflage and reduce predator detection (Lovell, Ruxton, Langridge, & Spencer, 2013). Similarly, animals might select habitat based on features that approximate natal habitats in which their parents were previously successful (Morse, 1999), thereby using information from their ecological environment to make space‐use decisions. Individuals may use information obtained from both their ecological and social environments to assess and access resources and the use of information from these sources represents two nonmutually exclusive strategies affecting access to resources.

Several hypotheses have been developed to identify the importance of ecological and social environments for sociality and space use. The conspecific attraction hypothesis (CAH) aims to provide a social explanation for animal space use (Stamps, 1988). The CAH suggests that animals use the presence of conspecifics as a positive cue for the quality of a resource patch and the probability of occupying a patch depends on whether it is already occupied by conspecifics. This can lead to spatially distributed aggregations of individuals, with seemingly suitable habitat left unoccupied because animals select resources based on attraction to conspecifics, rather than based on resource distribution directly (Ray, Gilpin, & Smith, 1991). The CAH has been demonstrated in a number of taxa. For example, orb‐web spiders (*Nephilengys cruentata*) were more likely to construct webs in areas where conspecifics already had webs, suggesting that spiders use the presence of conspecifics as an indirect cue for habitat quality (Schuck‐Paim & Alonso, 2001). The CAH posits that individuals will have stronger social associations (defined as the social circumstances in which interactions usually take place; Whitehead, 2008) when resource distribution is relatively patchy because animals rely on the location of conspecifics to find resource patches, and weaker social associations when resource distribution is relatively uniform (Fletcher, 2006). Conspecific attraction is therefore an important behavioral strategy which is related to animal space use in variable environments.

In some species, social association and space use is based on resource cues, which may be linked to biotic or abiotic features of the environment. The resource dispersion hypothesis (RDH) provides an environmental explanation for animal social association and space use (MacDonald, 1983). In the context of space use, the RDH suggests that when resource patches are spatiotemporally discrete, animals maintain territories or home ranges large enough to access sufficient resources to sustain energetic requirements (Johnson, Kays, Blackwell, & Macdonald, 2002). In environments where resources are distributed heterogeneously, there will be areas of local resource abundance that could be exploited by multiple individuals, with a low cost to all individuals that occupy and exploit a given resource patch. For example, savanna waterholes are heterogeneously distributed and animals often aggregate in large numbers at a single waterhole (Chamaillé‐Jammes, Fritz, Valeix, Murindagomo, & Clobert, 2008; Makin, Chamaillé‐Jammes, & Shrader, 2017). In the context of social association, the distribution of resources provides the underlying conditions for animals to share space (i.e., co‐occurrence: Farine, 2015; Spiegel, Leu, Sih, & Bull, 2016) independent of other benefits of group living, such as cooperation (Johnson et al., 2002). Although the RDH has been debated in the literature (Johnson & Macdonald, 2003; Revilla, 2003), there is evidence supporting resource dispersion as a proximate driver of animal social association and space use in a range of taxa (MacDonald & Johnson, 2015; Mcloughlin, Ferguson, & Messier, 2000). For example, in brown bears (*Ursus arctos*) home range size was higher for populations living in more seasonal environments (Mcloughlin et al., 2000). One prediction of the RDH is that animals will have larger home ranges when resource distribution is relatively patchy and smaller home ranges when resource distribution is relatively uniform, thus highlighting how animals use space according to the distribution of resources, as opposed to the presence of conspecifics. A logical extension of the RDH is that individuals should use cues from their physical environment (Van Moorter, Rolandsen, Basille, & Gaillard, 2016) to inform resource selection decisions, and these decisions should be made independent of the social environment.

In the context of integrating social and spatial processes, the presence of conspecifics (CAH) or familiarity with the distribution resources (RDH) may contribute to an individual's ability to access forage. Home range fidelity is a spatial process by which individuals return to previously used locations (Switzer, 1993), presumably because familiarity with the social and physical environments can improve fitness. In the context of the CAH, familiarity with conspecifics may enhance the attraction mechanism among individuals, thus reducing competition and increasing group‐level access to foraging resources (Wolf & Trillmich, 2007). By contrast, in the context of the RDH, familiarity with a particular location is predicted to enhance fine‐scale foraging success (Van Moorter et al., 2009). The degree of site fidelity in a population can also vary across spatial and temporal extents due to changes in predation or resource availability (van Beest, Vander Wal, Stronen, Paquet, & Brook, 2013; Schaefer, Bergman, & Luttich, 2000). Site fidelity should therefore vary based on whether individuals gain access to forage via social processes (CAH) or spatial processes (RDH). The CAH therefore posits that individuals should have low site fidelity, particularly when resources are heterogeneously distributed, because resource cues are obtained from conspecifics as opposed to environmental features, while the RDH posits that individuals should have high site fidelity, regardless of the presence of conspecifics.

Species that display fission‐fusion dynamics, where group size and composition vary through space and time, make a suitable system to examine ecological and social mechanisms driving space use and social organization. Caribou live in loosely associated fission–fusion societies with seasonal variation in both social organization and in the spatial distribution of forage on the landscapes they inhabit. Female caribou (*Rangifer tarandus*) tend to aggregate in groups during winter when forage resources are heterogeneously distributed and covered by snow (see Section 2; Barrette & Vandal, 1986). During summer, when the distribution of forage is, by comparison, relatively homogeneous, social groups dissolve and female caribou with calves tend to forage alone or in small groups (Stuart‐Smith, Bradshaw, Boutin, Hebert, & Rippin, 1997). For caribou in Gaspésie, Canada, dyads spent more time together in winter, when resources are relatively heterogeneous, compared to spring, summer, and autumn, when resources are relatively homogenous (Lesmerises, Johnson, & St‐Laurent, 2018). Predictions about the distribution of resources may be impractical to test in the field; therefore, we do not measure resource dispersion or abundance, but rather, our predictions are informed by the natural history and biology of caribou as they relate to seasonal differences in the access to forage (for examples see Bergerud, 1974; Briand, Ouellet, & Dussault, 2009; Hansen, Aanes, & Sæther, 2010; Rominger, Robbins, & Evans, 1996). We therefore considered winter and summer as proxies for heterogeneous and homogeneous spatial distribution of forage resources, respectively. We tested the RDH and CAH as nonmutually exclusive hypotheses to explain the spatial and social organization of female caribou. The CAH suggests that individuals use social processes to access forage patches, and, if female caribou behavior is driven by conspecific attraction (Table 1), we predicted:

P_1a_ Individual interannual site fidelity would be lower in winter when forage resources are distributed heterogeneously compared to summer when forage resource are distributed more homogenously. For example, we expect individuals to return to the same foraging sites in consecutive summers when they presumably rely less on social information, compared to winter where we expect site fidelity to be lower, as individuals are influenced by the presence of conspecifics.

P_2a_ Home range overlap among individuals would be higher in winter when caribou rely more on social processes and the presence of conspecifics compared to summer. We expect individuals to have relatively high home range overlap in winter because sharing home ranges is a necessary prerequisite for social association or interaction.

P_3a_ Individual social association would be higher in winter when caribou rely more on social processes and the presence of conspecifics compared to summer, and social associations would be higher than randomly generated social associations in each season, respectively.

**Table 1:** Predictions of conspecific attraction (CAH) and resource dispersion (RDH) hypotheses with associated conclusions

Variable	Hypothesis	Predictions and outcomes	Associated conclusions	Result of our study
Interannual site fidelity	CAH	(1a) Interannual site fidelity lower in winter compared to summer	(1a) CAH supported	(1a) Yes
		(2a) No difference in interannual site fidelity between seasons	(2a) Null (no support for CAH or RDH)	(2a) No
Home range overlap	CAH	(1b) Home range overlap higher in winter compared to summer	(1b) CAH supported	(1b) Yes
		(2b) No difference in home range overlap between seasons	(2b) Null (no support for CAH or RDH)	(2b) No
Social association	CAH and RDH	(1c) Social association higher in winter compared to summer	(1c) CAH supported	(1c) Yes
		(2c) No difference in social association between seasons	(2c) Null, but RDH supported	(2c) No
		(3c) Observed social association differs from randomly generated social association in both seasons	(3c) CAH supported	(3c) Yes, winter
		(4c) No difference between observed and random social association within seasons	(4c) Null, but RDH supported	(4c) Yes, summer
Home range area	RDH	(1d) No correlation between home range area and social association in both seasons	(1d) RDH supported	(1d) No
		(2d) Positive correlation between home range area and social association in both seasons	(2d) Null (no support for CAH or RDH)	(2d) Yes, both seasons
		(3d) Home range area larger in winter compared to summer	(3d) RDH supported	(3d) Yes
		(4d) No difference in home range area size between seasons	(4d) Null (no support for CAH or RDH)	(4d) No

Note

Note, in some cases the null hypothesis supports either the CAH or RDH, but in other cases the null hypothesis may be driven by unmeasured phenomena.

The RDH suggests that individuals use spatial cues about the quality of habitats to find patches of forage, and, if female caribou behavior is driven by the distribution of resources (Table 1), we predicted:

P_1b_ Individual social associations will not differ from randomly generated social associations within each season, because social association should occur due to random processes whereby individuals share space by chance, as opposed to by preference.

P_2b_ No correlation between individual home range area and social association because home range size is expected to be related to resource dispersion, while social association is expected to be related to abundance of resource patches.

P_3b_ Individual home range area will be larger in winter when resource distribution is relatively heterogeneous compared to summer because individuals will require a larger area to acquire resources when resource distribution is heterogeneous. Although home range area is influenced by a variety of seasonally dependent factors, including maternal status and calf mobility, the distribution of resources in winter should be the primary factor driving larger home ranges during this season.

## 2 METHODS

### 2.1 Study area and resource distribution

Newfoundland is an island off eastern Canada (47°44′N, 52°38′W to 51°44′N, 59°28′W) with a humid‐continental climate and persistent precipitation throughout the year (Environment and Climate Change Canada). Caribou forage primarily on lichen, grasses, sedges, and other deciduous browse (Bergerud, 1974; Mahoney & Virgl, 2003). Forage resources for caribou change between the seasons due to accessibility. During summer (July–September), the absence of snow yields a relatively homogeneous distribution and higher abundance of vegetation compared to winter. During winter (January–March), when the landscape is covered by snow, access to vegetation becomes limited. From 2006–2012, the average monthly snowfall in winter (January–March) was 91.5 cm (*SD* = 55.7 cm), and the average monthly depth of snow on the ground was 43.9 cm (*SD* = 32.7 cm, min = 0.2 cm, max = 117.6 cm; Environment & Climate Change Canada, 2017). In Newfoundland, wolves (*Canis lupus*) are extirpated, so coyotes (*Canis latrans*) and black bears (*Ursus americanus*) are the primary predators of caribou (Bastille‐Rousseau, Schaefer et al., 2016b). Coyotes and black bears are responsible for the majority of mortalities of neonate caribou calves (Bastille‐Rousseau, Schaefer et al., 2016b), although predation can still occur after this period (Lewis & Mahoney, 2014). By contrast, although predation by coyotes or black bears on adult female caribou is possible, it is relatively rare.

To access forage in the winter, caribou dig holes in the snow, termed craters (Bergerud, 1974). Caribou in Newfoundland tend to dig craters in locations where snow depth is relatively shallow (~30–60 cm deep), such as hillsides or hummocks (Bergerud, 1974). As a result, caribou cannot access all subnivean forage and tend to occupy and reuse craters once they are established. The average area of craters dug by caribou in Newfoundland was 0.41 m^2^ (*SD* = 0.48; Mayor, Schaefer, Schneider, & Mahoney, 2009) and crater density, which varies based on snow condition, depth, and local caribou density, can range from 366 to 1,980 craters/ha (Bergerud, 1974; Pruitt, 1959); there is therefore considerably less access to forage than when the landscape is snow‐free. The distribution of craters on the landscape is heterogeneous, and we consider access to vegetation in winter to be highly variable among individual caribou.

### 2.2 Location data

We used GPS location data collected from three caribou herds in Newfoundland: Middle Ridge (2009–2013), Topsails (2007–2011), and Fogo Island (2016–2018). The population density of the three caribou herds was relatively stable during the period of our study (Bastille‐Rousseau, Schaefer, Mahoney, & Murray, 2013). Adult female caribou from all herds were immobilized and fitted with global positioning system (GPS) collars (Lotek Wireless Inc., Newmarket, ON, Canada, GPS4400M collars, 1,250 g) as described by Schaefer and Mahoney (2013). Collars were deployed on individual caribou for one to three years, and collars were often redeployed on the same individuals for up to five years. Collars were programmed to collect location fixes every 1 or 2 hr, depending on the season, herd, and year. Prior to analyses, we removed all erroneous and outlier GPS fixes following Bjørneraas, Moorter, Rolandsen, and Herfindal (2010). To assess seasonal differences in our response variables, we subset GPS fixes into discrete 48‐day periods that reflect winter (15 January–3 March) and summer (15 July–1 September). We chose these dates for two reasons: (a) to ensure resource distribution was relatively predictable within season (heterogeneous during the winter and homogeneous during the summer); and (b) to ensure that caribou space use in winter and summer, respectively, was not impacted by behaviors during adjacent seasons (i.e., calving season: typically May–June; mating season: typically September–October; Bastille‐Rousseau, Rayl et al., 2016a). We did not collar all female caribou in the herds, however, we assumed that our sample of collared animals was random. Although associations between collared and uncollared animals were unrecorded, we assumed that our networks (see below) were unbiased representations of the relative degree of social association among all caribou.

### 2.3 Social network analysis

We used R package *spatsoc* (version 0.1.6, Robitaille, Webber, & Vander, 2018) in R version 1.1.383 (R Core Team, 2017) to generate proximity‐based social networks (PBSNs) from GPS telemetry data. We generated social networks for each herd in each season based on proximity of GPS fixes for individual caribou. We assumed association between two individuals if simultaneous GPS fixes (i.e., recorded within 5 min of each other) were within 50 m of one another (Lesmerises et al., 2018). We represented individuals in our networks by nodes and associations between individuals were represented by edges.

We applied the “chain rule,” where each discrete spatiotemporal GPS fix was buffered by 50 m, and we considered individuals in the same group if 50 m buffers for two or more individuals were contiguous, even if some individuals within the buffer were not within 50 m of one another. Group assignment based on the chain rule has commonly been applied to gregarious mammals (Gero, Gordon, & Whitehead, 2013), including reindeer (*R. tarandus)* in Fennoscandia (Body, Weladji, Holand, & Nieminen, 2015). We weighted edges of social networks by the strength of association between dyads of caribou using the simple ratio index (SRI; Cairns & Schwager, 1987):

$$\text{SRI}=\frac{x}{x+y_{\mathrm{AB}}+y_{A}+y_{B}}$$

where *x* is the number of fixes where individuals A and B were within 50 m of each other, *y_A_* is the number of fixes from individual A when individual B did not have a simultaneous fix, *y_B_* is the number of fixes from individual B when individual A did not have a simultaneous fix, and *y_AB_* is the number of simultaneous fixes from individuals A and B that were separated by >50 m (Farine & Whitehead, 2015). Social groups were designated if two or more individuals occurred within 50 m of one another at any given time point. We generated social networks with the *igraph* package in R, version 1.2.2 (Csárdi & Nepusz, 2006). For each network, we calculated graph strength, defined as the sum of the edge weights for each individual in each network. We considered graph strength generated from PBSNs as an index of sociality (i.e., social strength).

We compared observed social strength values to randomly generated social strength values. We randomized PBSNs based on the raw data stream (i.e., GPS fixes) to reduce potential for type II error typically associated with node‐based permutations (Farine, 2014). Following Spiegel et al. (2016), we reordered daily GPS movement trajectories for each individual while maintaining the temporal path sequence within each time block. This technique is a robust network randomization procedure for GPS data because: (a) it maintains the spatial aspects of an individual's movement; and (b) by randomizing movement trajectories of individuals independent of one another, temporal dependencies of movement are decoupled (Spiegel et al., 2016). We repeated this procedure 1,000 times for each network (i.e., year‐by‐season‐by‐herd combination) by regenerating PBSNs and calculating social strength at each iteration. We then extracted the mean graph strength value across the 1,000 randomly generated networks for each individual in each network and paired this value with the observed social strength value for the same individual in the same network. We also assessed whether observed values of social strength differed from randomized values of social strength using a mixed modeling framework (i.e., year‐by‐season‐by‐herd combination).

### 2.4 Social network randomization procedure

In addition to comparing observed to random network metrics at the individual level, we also compared the observed and random values of social graph strength. In our primary randomization analysis, we followed the randomization procedure outlined by Spiegel et al. (2016). Due to the nature of GPS relocation data and the possibility of individual differences in movement trajectories (Spiegel et al., 2016), this randomization procedure segments movement trajectories into temporally discrete units (e.g., daily or weekly) and shuffles the order of trajectories (e.g., day 1 and day 2 may be swapped) for each individual. We used daily trajectories and shuffled them for each individual, while maintaining the temporal sequence of GPS relocations within each trajectory (for details see Spiegel et al., 2016). We repeated this procedure 1,000 times for each network (i.e., year‐by‐season‐by‐herd combination) by regenerating social networks and calculating social strength at each iteration. We then extracted the graph strength values across the 1,000 randomly generated networks for each individual in each network and assessed the effects of season on randomly generated values of graph strength in a mixed modeling framework (Farine & Whitehead, 2015). Models included randomized graph strength for each individual as the response variable with season (summer or winter) as a fixed effect. We included year as a random effect as well as individual identification nested within herd (Middle Ridge, Topsails, or Fogo Island herds). We extracted coefficient estimates for the model intercept as well as for season from each of the 1,000 models and generated a random distribution of estimates which we compared to the observed estimates for the intercept and for season. We considered observed estimates significantly different from random distributions of estimates if they fell outside the 95% confidence intervals of the distribution.

### 2.5 Home range area and overlap

We estimated caribou home ranges using the area of the 95% isopleths from fixed kernel density estimates (KDE; Worton, 1989) for each individual in each season with the *href* smoothing parameter in the *adehabitatHR* package version 0.4.15 in R (Calenge, 2006). We first estimated home range area for all individual‐by‐year‐by‐season combinations and compared home range area to social association across seasons (see below). We then estimated home range overlap with the utilization distribution overlap index (UDOI; Fieberg & Kochanny, 2005), where higher dyadic values of UDOI represent a greater proportion of overlap and lower values represent lower proportion of overlap. Based on pairwise combinations of UDOI, we generated networks, hereafter spatial networks, with edges weighted by dyadic UDOI values and calculated graph strength (the sum of edge weights for each individual in the spatial network) as a measure that captures the degree to which an individual's home range overlaps with that of other collared individuals (hereafter, spatial graph strength). We also quantified within‐individual consistency of seasonal home range use (i.e., site fidelity) by comparing season‐specific UDOI estimates for each individual across years. We conducted all home range analyses with the *adehabitatHR* package in R (Calenge, 2006).

### 2.6 Statistical analyses

Prior to statistical analysis, we visually assessed all variables for outliers. Three individuals had extremely large summer home ranges (>4,000 km^2^), so we removed these individuals from all subsequent analyses. We log‐transformed all variables for subsequent analyses to ensure that residuals were normally distributed. We evaluated our predictions using linear mixed models in the *lme4* package in R (Bates et al., 2015). We ran four separate models: one with each of site fidelity (P_1a_ for CAH), spatial graph strength (P_2a_ for CAH), social graph strength (P_3a_ for CAH and P_1b_ for RDH), and home range area (P_2b_ and P_3b_ for RDH) as response variables (see Table 1). Each model included season (summer or winter) as a fixed effect and individual identification nested within herd (Middle Ridge, Topsails, or Fogo Island herds) as well as year as random effects. For the interannual site fidelity model, we modified year to account for the pair of years across which fidelity was being estimated (e.g., fidelity from 2007 to 2008) and we incorporated this variable as a random effect. For the social strength model, we paired the observed value of social strength with a randomly generated value of social strength (see above) for each individual and we incorporated an additional fixed effect (i.e., observed vs. random) as an interaction with season to test for potential within‐season differences between random and observed values of social strength. For the home range area model, we included observed social graph strength as a predictor for home range area.

## 3 RESULTS

We used 87 individual caribou from three herds (*n* = 41 for Middle Ridge herd, *n* = 27 for Topsails herd, and *n* = 19 for Fogo Island herd) in our analyses, for a total of 370 unique caribou‐season‐years (Table 2). For the Middle Ridge herd, we obtained an average of 563 (*SD* = 340) GPS locations per caribou in summer, and an average of 463 (*SD* = 336) GPS locations per caribou in winter. For the Topsails herd, we obtained an average of 556 (*SD* **= **19) GPS locations per caribou in summer, and an average of 504 (*SD* = 142) GPS locations per caribou in winter. For the Fogo Island herd, we obtained an average of 522 (*SD* **= **101) GPS locations per caribou in summer, and an average of 556 (*SD* = 12) GPS locations per caribou in winter. Based on these GPS locations, we observed a total of 3,797 social groups of two or more GPS collared individuals. On average, we observed 328 (*SD* = 579) groups per winter and 15 (*SD* = 21) groups per summer.

**Table 2:** Average number (*SD*) of individual caribou (*Rangifer tarandus*) in each network, average social and spatial graph strength (*SD*), and average home range area (*SD*) for Middle Ridge (2009–2013), Topsails (2007–2011), and Fogo Island (2016–2018) caribou herds in Newfoundland, Canada

	Middle Ridge		Topsails		Fogo Island	
	Winter	Summer	Winter	Summer	Winter	Summer
Number of individuals	22.3 (3.3)	19.0 (3.5)	16.2 (2.7)	15.5 (2.1)	8.5 (2.1)	13.5 (2.1)
Social strength^a^	0.017 (0.024)	0.001 (0.002)	0.014 (0.02)	0.001 (0.003)	0.56 (0.53)	0.007 (0.01)
Spatial strength^b^	3.63 (2.30)	0.27 (0.50)	1.17 (1.20)	0.18 (0.35)	2.77 (1.79)	0.24 (0.23)
Home range area (km^2^)^c^	495 (376)	279 (431)	334 (448)	154 (323)	50.1 (30.1)	17.5 (24.0)

a We calculated average social strength as the sum of weighted edges based on social networks.

b We calculated average spatial strength as the sum of weighted edges based on home range overlap.

c We estimated average seasonal home range area using the 95% isopleth of the kernel density estimator (Worton, 1989).

**Figure 1:**
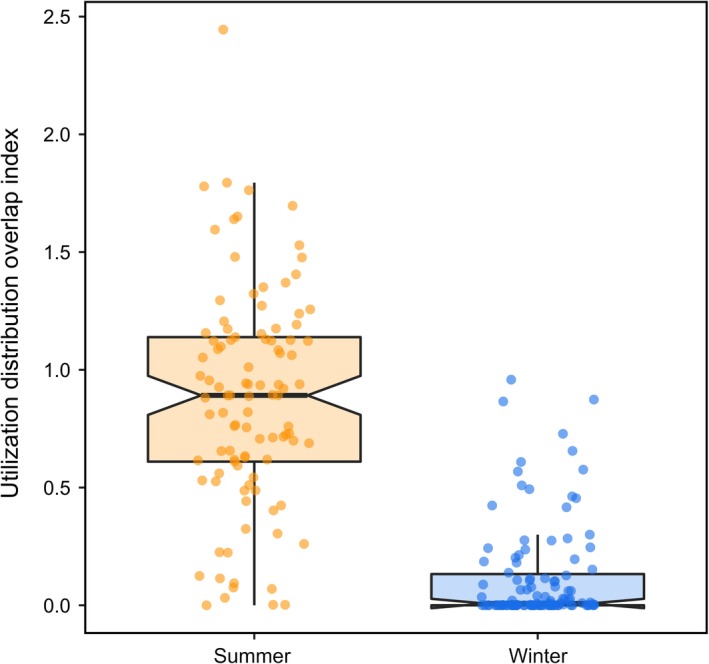
Interannual site fidelity, estimated as within‐individual utilization distribution overlap index (UDOI) values, from year to year (e.g., 2007–2008) during summer (orange) and winter (blue) for individual woodland caribou (*Rangifer tarandus*) in Newfoundland, Canada. Higher UDOI values reflect stronger home range overlap from year to year within a given season. Points show the distribution of data, thick dark lines represent the median, upper and lower edges of each box represent the interquartile range (25% and 75% of data), notches represent the qualitative difference in median in each season, and whiskers represent the upper and lower quantiles (2.5% and 97.5% of data)

**Figure 2:**
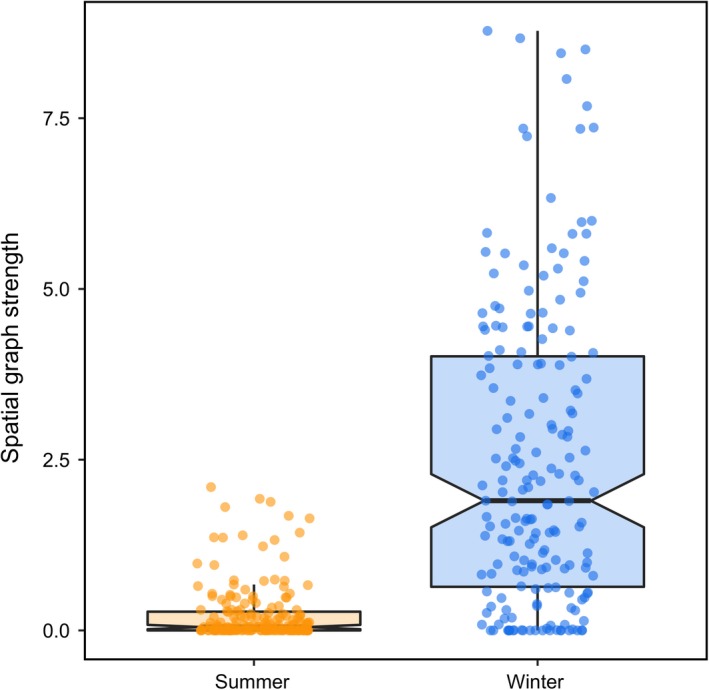
Spatial graph strength of individual woodland caribou (*Rangifer tarandus*) across three herds in Newfoundland, Canada during summer (orange) and winter (blue). We measured spatial graph strength as the sum of weighted edges based on home range overlap networks and higher values of spatial graph strength represent individuals that had higher home range overlap with conspecifics. Points show the distribution of data, thick dark lines represent the median, upper and lower edges of each box represent the interquartile range (25% and 75% of data), notches represent qualitative difference in median in each season, and whiskers represent the upper and lower quantiles (2.5% and 97.5% of data)

**Figure 3:**
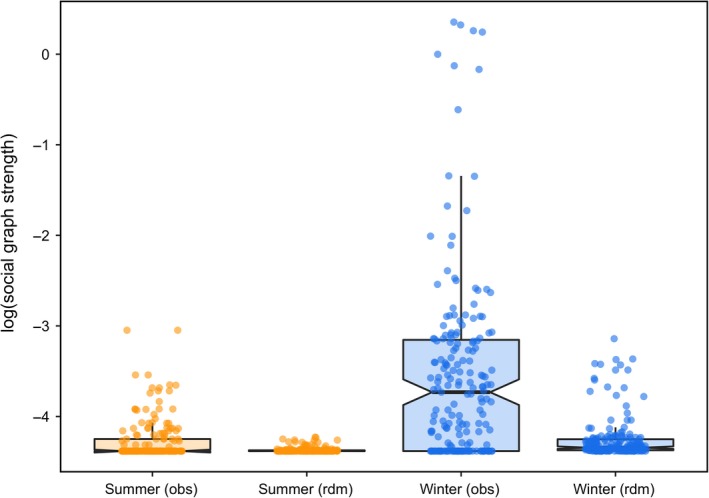
Social graph strength of individual woodland caribou (*Rangifer tarandus*) across three herds in Newfoundland, Canada during summer (orange), and winter (blue). We calculated social graph strength as the sum of weighted edges based on proximity‐based social networks (denoted as “obs”). We calculated social graph strength for random networks (denoted as “rdm”) by reordering GPS movement trajectories of individual caribou across 1,000 iterations. Points show the distribution of data, thick dark lines represent the median, upper and lower edges of each box represent the interquartile range (25% and 75% of data), notches represent qualitative difference in median in each season, and whiskers represent the upper and lower quantiles (2.5% and 97.5% of data). Note, social graph strength is log‐transformed for ease of interpretation

Taken together, our models support the CAH in winter, but not summer. Caribou had low interannual site fidelity to their winter ranges (Figure 1; Table 3), indicating that individuals used different seasonal ranges in consecutive years. Individual caribou also had higher home range overlap (Figure 2; Table 3) and social association in winter compared to summer (Figure 3), thus providing empirical support for the CAH in winter.

**Table 3:** Summary of four models testing the effects of season and herd on interannual site fidelity, spatial graph strength, social graph strength, and home range area of caribou (*Rangifer tarandus*) in Newfoundland, Canada

Interannual site fidelity model (P_1a_, CAH)	β (± *SE*)	*t*‐value	*p*‐value^a^
Intercept	–0.36 ± 0.17	–2.44	**0.01**
Season (winter)	–2.73 ± 0.17	–15.8	**<0.001**
Random variables	Variance (±*SD*)		
Herd:ID	6.8 × 10^−15^ ± 8.2 × 10^−8^		
Herd	0.02 ± 0.13		
Year^b^	6.3 × 10^−14^ ± 2.5 × 10^−7^		
Residual	1.54 ± 1.24		

Spatial strength model (P_2a_, CAH)	β (±*SE*)	*t*‐value	*p*‐value^a^
Intercept	–2.78 ± 0.57	–4.87	**<0.001**
Season (winter)	2.88 ± 0.15	19.3	**<0.001**
Random variables	Variance (±*SD*)		
Herd:ID	0.94 ± 0.97		
Herd	0.82 ± 0.90		
Year	0.26 ± 0.51		
Residual	1.80 ± 1.34		

Social strength model (P3a, CAH; P1b, RDH)	β (±*SE*)	*t*‐value	*p*‐value^a^
Intercept	–4.21 ± 0.19	–21.7	**<0.001**
Network type (rdm)	–0.10 ± 0.05	–2.00	**0.045**
Season (winter)	0.77 ± 0.05	15.6	**<0.001**
Network type (rdm) × Season (winter)	–0.61 ± 0.07	–9.04	**<0.001**
Random variables	Variance (±*SD*)		
Herd:ID	0.01 ± 0.11		
Herd	0.09 ± 0.31		
Year	0.05 ± 0.22		
Residual	0.21 ± 0.46		

Home range area model (P_2b_ & P_3b_, RDH)	β (±*SE*)	*t*‐value	*p*‐value^a^
Intercept	14.2 ± 2.35	6.0	**<0.001**
log(social strength)	1.67 ± 0.53	3.2	**0.004**
Season (winter)	–3.86 ± 2.24	–1.8	0.07
log(social strength) × season (winter)	–1.16 ± 0.50	–2.3	**0.02**
Random variables	Variance (±*SD*)		
Herd:ID	0.58 ± 0.76		
Herd	1.94 ± 1.39		
Year	0.16 ± 0.40		
Residual	1.678 ± 1.33		

Notes

Model results are delineated by rows with gray shading that indicate the response variable for each model set as well as the corresponding hypothesis (CAH: conspecific attraction hypothesis or RDH: resource dispersion hypothesis) and predictions.

a Bold font indicates statistical significance (α < 0.05).

b Year for the site fidelity model accounts for the pair of years across which we compared site fidelity.

**Figure 4:**
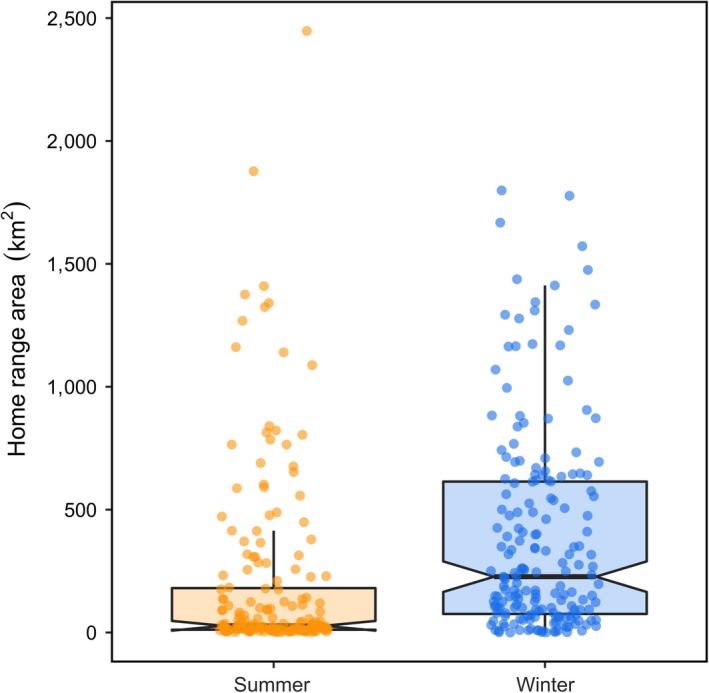
Home range area (km^2^) of individual woodland caribou (*Rangifer tarandus*) across three herds in Newfoundland, Canada during summer (orange), and winter (blue). We estimated home range area using the 95% isopleth of the kernel density estimator (Worton, 1989). Points show the distribution of data, thick dark lines represent the median, upper and lower edges of each box represent the interquartile range (25% and 75% of data), notches represent qualitative difference in median in each season, and whiskers represent the upper and lower quantiles (2.5% and 97.5% of data)

**Figure 5:**
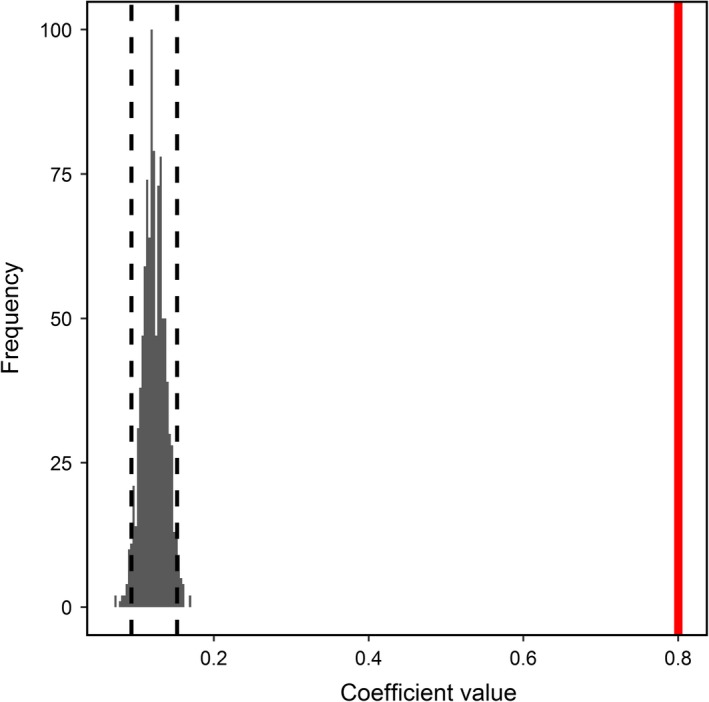
Comparison of randomly generated coefficient estimates for a linear mixed effects model testing the effects of season (winter represented and summer as the reference category) on social graph strength with individual identity nested within herd and year as random effects. This randomization highlights how social graph strength is higher in winter compared to summer and this relationship is nonrandom. Note, the vertical red line represents the coefficient estimate from the observed model presented in Table 3 and dashed lines represent 95% confidence intervals around the randomly generated distribution

By contrast, our models provide mixed support the RDH in summer, but no support in winter. Specifically, we observed that social association did not differ relative to random in summer, whereas in winter, social association differed significantly from random (Figure 3; Table 3), thus providing some empirical support for the RDH in summer. By contrast, we observed weak correlations between home range area and observed social association in both seasons (Table 3), while home range areas were similar across seasons (Figure 4; Table 3), thus failing to support the RDH in either season. Based on our randomization procedure, all individual measures of social strength differed from randomly generated distributions of social strength (Figure 5).

## 4 DISCUSSION

We tested the resource dispersion (RDH) and conspecific attraction (CAH) hypotheses as drivers of variation in space use and social association for caribou. We found support for the CAH in winter and some support for the RDH in summer. Our findings suggest that caribou social association varies across seasons, which could either be an outcome of seasonal variation in foraging behavior or, alternatively, could be a driver of social information about forage opportunities in winter. Either way, our findings contribute to the growing body of literature that highlights the link between social association and space use in caribou (Lesmerises et al., 2018). We also highlight seasonal variation in how access to forage can be used as an indirect test to better understand the relationship between social processes and foraging in caribou. Because foraging behavior tends to be more flexible as environments become more seasonal, we expected greater spatiotemporal variation in the relationship between individual social behavior and space use (Webber & Vander Wal, 2018). Our results provide season‐specific support for the CAH and, by extension, we highlight links between social association and space use in caribou.

We found support for the CAH in winter, when resources were heterogeneously distributed. Interannual site fidelity was lower in winter than summer, presumably because the distribution of craters on the landscape is less predictable and changes from year to year, whereas in summer, the distribution of resources is relatively similar from year to year. Low interannual site fidelity in winter could be related to social processes that inform an individual's understanding of the distribution of resources, but it could also be related more directly to the distribution of resources. In summer, high interannual site fidelity is likely related to habitat quality (Schaefer & Mahoney, 2013), but could also be related to maternal status as familiarity with resource availability and abundance is an important predictor of reproductive success (Lafontaine, Drapeau, Fortin, & St‐Laurent, 2017). Previous studies of caribou have also observed higher fidelity to summer ranges relative to winter ranges (Faille et al., 2010; Lafontaine et al., 2017; Schaefer & Mahoney, 2013; Wittmer, McLellan, & Hovey, 2006). These patterns have also been observed in other species where resource distribution varies seasonally. For example, red knots (*Calidrus c. canutus*) exhibited high interannual site fidelity when forage was predictable and homogenously distributed (Leyrer, Spaans, Camara, & Piersma, 2006). One interpretation of our results is that, because interannual site fidelity was relatively low in winter, caribou may be more likely to use social cues to locate forage. Alternatively, it is possible that caribou deplete resource patches in a given winter and so do not return to the same depleted areas in consecutive years.

We also found support for the CAH in the form of high home range overlap and social association in winter when resources were more heterogeneously distributed. One interpretation of these findings is that caribou use social cues from conspecifics to locate accessible forage during winter. Our observation that multiple caribou used the same space at the same time can be interpreted at two scales: the broad feeding area scale and the local crater scale. The feeding area scale reflects aggregations of craters within a 150 m^2^ area and separated from neighboring aggregations by at least 50 m (Mayor et al., 2009). Caribou feeding areas in Newfoundland tend to have softer, shallower snow and are richer in winter forage relative to the range of the entire herd (Mayor et al., 2009). Thus, caribou may use the presence of conspecifics to locate feeding areas in winter when the average home range size was up to 500 km^2^ and would have contained many feeding areas. Craters generally only support a small number of feeding caribou at a time, and many social associations among caribou in winter presumably involve individuals displacing other individuals to gain access to craters (Barrette & Vandal, 1986). Similarly, larger social group sizes of bison (*Bison bison*) have been linked with increased likelihood of locating and consuming cryptic forage under snow (Fortin & Fortin, 2009). These observations, in combination with our finding of higher home range overlap and social association in winter, suggest that caribou may observe the feeding behavior of conspecifics and use this as a signal of resource presence.

We found some support for the RDH, particularly in summer. Caribou social associations were not different from random in summer, and home ranges were smaller in summer compared to winter, supporting the RDH. By contrast, we observed weak correlations between home range area and social association in both seasons, a result which does not support the RDH (Table 1). This relationship, however, is notoriously difficult to quantify and interpret (Robertson, Palphramand, Carter, & Delahay, 2015), largely due to the wide variety of factors that can influence home range size (Börger, Dalziel, & Fryxell, 2008). We observed considerable variation in the size of individual home ranges, particularly in summer. Variation in home range size may be explained by other factors, including maternal status of females. Females without calves are not restricted by the slower movement speed of their calf (Bonar, Ellington, Lewis, & Vander Wal, 2018; DeMars, Auger‐Méthé, Schlägel, & Boutin, 2013), and often have very large home ranges in summer. It is also possible that the distribution of anthropogenic disturbances, such as logging cutovers, could also influence the high degree of variation in home range size we observed in summer (Faille et al., 2010; MacNearney et al., 2016; Schaefer & Mahoney, 2007). The correlation we observed between home range area and social association therefore does not necessarily preclude the role of resource dispersion and abundance in explaining caribou home range size. We interpret these findings as weak support for the RDH in summer when resource distribution is relatively homogeneous compared to winter.

A potential alternative hypothesis is that animal space use and social association are driven by predation risk. In many gregarious ungulates, social aggregation is commonly cited as an antipredator behavior (Creel, Schuette, & Christianson, 2014; Lingle, 2001). Predation can affect behavioral strategies of prey through nonconsumptive effects, which are explicitly associated with predation risk (Orrock et al., 2008), a process which can also affect the spatial distribution of prey (Moll et al., 2017). In summer, it could be that the risk of calf predation by coyotes or black bears (Bastille‐Rousseau, Schaefer et al., 2016b) represents an alternative mechanism explaining variation in home range size. Although no data exist on encounter rates among caribou and their predators in our study area, for females with calves‐at‐heel, predation risk during summer when calves are a few months old could suggest that some female caribou with very large home ranges move longer distances after encountering predators. Evidence also exists suggesting that caribou avoid risky habitat (Bastille‐Rousseau, Rayl et al., 2016a) and that caribou dyads are more likely to stay together when risk of predation is high, especially in winter (Lesmerises et al., 2018). Taken together, predation is likely an important driver of both social association and space use and although we were unable to incorporate aspects of predation in our study, we encourage future studies to simultaneously model effects of predation on social association and space use in caribou.

Our results also contribute to the ongoing discussion on the relationship between spatial structure of the environment and social organization (Castles et al., 2014; Farine, 2015). It is possible that social associations are simply a by‐product of individuals sharing space, rather than preferentially associating, suggesting social aggregations may in fact reflect co‐occurrence (i.e., animals that share space, but do not have direct social assocation sensu Farine, 2015; Spiegel et al., 2016). Castles et al. (2014) suggested that social interaction networks were not correlated with proximity‐based networks and that networks generated based on different behaviors should not be used as proxies for one another. In contrast, Farine (2015) suggested that regardless of correlations between network types, proximity among individuals, or co‐occurrence, remains an important form of social behavior, and therefore a relevant means to construct social networks. While we do not explicitly test for relationships between social interactions and co‐occurrence, we posit that networks constructed using GPS telemetry data may provide important insight into this issue. Specifically, the randomization procedure proposed by Spiegel et al. (2016) which we have adopted here, decouples social association from space use by randomizing movement trajectories within (as opposed to between) individuals. If observed social association differs from random social association, individuals presumably associate nonrandomly. Thus, if observed social association does not differ from random, we expect co‐occurrence represents a valuable type of social behavior, as suggested by Farine (2015).

We empirically tested the CAH and the RDH in caribou and our findings suggest that space use and social association vary across seasons, where the dispersion of resources was a driving factor in summer and conspecific attraction was important in winter. Social behavior varies among individuals within populations and across species, ranging from relatively solitary to highly gregarious. Seasonal variation in social association and space use is also important to consider along this continuum because it highlights the plasticity of animal behavior and the ability of many species, populations, and individuals to adapt to seasonal variation in resource access. As caribou populations continue to decline in Canada and around the world (Mallory & Boyce, 2017; Vors & Boyce, 2009), it is increasingly likely that conspecific attraction and the use of social processes to gain information about resources (Lesmerises et al., 2018) will be compromised because of declining population density. The downstream consequences could impact individuals by negatively affecting survival and reproduction, which could further compound the issue of declining populations. For caribou, we expect reduced population density would be most impactful in winter given this is the period when resources are most limiting and that we found higher levels of social association in winter. We suggest that future studies of caribou socioecology assess fine‐scale social interactions within and between foraging sites in winter to determine the role of conspecific attraction in the winter foraging ecology of caribou.

## ETHICAL STATEMENT

Funding for this study was provided by a Vanier Canada Graduate Scholarship to QMRW, a National Sciences and Engineering Research Council (NSERC) Post‐Doc Fellowship to ELK, a NSERC Post‐Graduate Scholarship to MPL, and a NSERC Discovery Grant to EVW. All animal capture and handling procedures were consistent with the American Society of Mammologists guidelines (Sikes & Gannon, 2011) and were approved by Memorial University Animal Use Protocol No. 20152067.

## CONFLICT OF INTEREST

The authors declare that they have no conflict of interest.

## AUTHORS' CONTRIBUTIONS

MP and QMRW contributed equally to all aspects of the study, including conceiving the study, processing data and conducting statistical analysis, and drafting the manuscript; ELK helped conceive the study and revised the manuscript; MPL and ALR helped with data processing and management and revised the manuscript; EVW developed the research program, helped conceive the study, and revised the manuscript.

## Data Availability

All code used for all statistical analysis and figures are available from Zenodo: https://zenodo.org/record/2580325 and data are available for Dryad Digital Repository: https://doi.org/10.5061/dryad.vd7d8v6

## References

[ece35071-bib-0001] Barrette, C. , & Vandal, D. (1986). Social rank, dominance, antler size, and access to food in snow‐bound wild woodland caribou. Behaviour, 97, 118–146. 10.1163/156853986X00342

[ece35071-bib-0002] Bastille‐Rousseau, G. , Rayl, N. D. , Ellington, E. H. , Schaefer, J. A. , Peers, M. J. L. , Mumma, M. A. , … Murray, D. L. (2016a). Temporal variation in habitat use, co‐occurrence, and risk among generalist predators and a shared prey. Canadian Journal of Zoology, 94, 191–198.

[ece35071-bib-0003] Bastille‐Rousseau, G. , Schaefer, J. A. , Lewis, K. P. , Mumma, M. A. , Ellington, E. H. , Rayl, N. D. , … Murray, D. L. (2016b). Phase‐dependent climate‐predator interactions explain three decades of variation in neonatal caribou survival. Journal of Animal Ecology, 85, 445–456. 26529139 10.1111/1365-2656.12466

[ece35071-bib-0004] Bastille‐Rousseau, G. , Schaefer, J. A. , Mahoney, S. P. , & Murray, D. L. (2013). Population decline in semi‐migratory caribou (*Rangifer tarandus*): Intrinsic or extrinsic drivers? Canadian Journal of Zoology, 91, 820–828.

[ece35071-bib-0005] Bates, D. , Maechler, M. , Bolker, B. , Walker, S. , Christensen, R. H. B. , Singmann, H. , … Grothendieck, G. (2015). Fitting linear mixed‐effects models using lme4. Journal of Statistical Software, 67, 5133–48.

[ece35071-bib-0006] Bergerud, A. T. (1974). Relative abundance of food in winter for Newfoundland caribou. Oikos, 25, 379–387. 10.2307/3543960

[ece35071-bib-0007] Bjørneraas, K. , Van Moorter, B. , Rolandsen, C. M. , & Herfindal, I. (2010). Screening global positioning system location data for errors using animal movement characteristics. Journal of Wildlife Management, 74, 1361–1366. 10.1111/j.1937-2817.2010.tb01258.x

[ece35071-bib-0008] Body, G. , Weladji, R. B. , Holand, O. , & Nieminen, M. (2015). Fission‐fusion group dynamics in reindeer reveal an increase of cohesiveness at the beginning of the peak rut. Acta Ethologica, 18, 101–110. 10.1007/s10211-014-0190-8

[ece35071-bib-0009] Bonar, M. , Ellington, E. H. , Lewis, K. P. , & Vander Wal, E. (2018). Implementing a novel movement‐based approach to inferring parturition and neonate calf survival. PLoS ONE, 13, e0192204. 29466451 10.1371/journal.pone.0192204 PMC5821316

[ece35071-bib-0010] Börger, L. , Dalziel, B. D. , & Fryxell, J. M. (2008). Are there general mechanisms of animal home range behaviour? A review and prospects for future. Ecology Letters, 11, 637–650. 18400017 10.1111/j.1461-0248.2008.01182.x

[ece35071-bib-0011] Briand, Y. , Ouellet, J. , & Dussault, C. (2009). Fine‐scale habitat selection by female forest‐dwelling caribou in managed boreal forest: Empirical evidence of a seasonal shift between foraging opportunities. Ecoscience, 16, 330–340.

[ece35071-bib-0012] Cairns, S. J. , & Schwager, S. J. (1987). A comparison of association indices. Animal Behaviour, 35, 1454–1469. 10.1016/S0003-3472(87)80018-0

[ece35071-bib-0013] Calenge, C. (2006). The package “adehabitat” for the R software: A tool for the analysis of space and habitat use by animals. Ecological Modelling, 197, 516–519. 10.1016/j.ecolmodel.2006.03.017

[ece35071-bib-0014] Castles, M. , Heinsohn, R. , Marshall, H. H. , Lee, A. E. G. , Cowlishaw, G. , & Carter, A. J. (2014). Social networks created with different techniques are not comparable. Animal Behaviour, 96, 59–67. 10.1016/j.anbehav.2014.07.023

[ece35071-bib-0015] Chamaillé‐Jammes, S. , Fritz, H. , Valeix, M. , Murindagomo, F. , & Clobert, J. (2008). Resource variability, aggregation and direct density dependence in an open context: The local regulation of an African elephant population. Journal of Animal Ecology, 77, 135–144. 10.1111/j.1365-2656.2007.01307.x 17986249

[ece35071-bib-0016] Creel, S. , Schuette, P. , & Christianson, D. (2014). Effects of predation risk on group size, vigilance, and foraging behavior in an African ungulate community. Behavioral Ecology, 25, 773–784. 10.1093/beheco/aru050

[ece35071-bib-0017] Csárdi, G. , & Nepusz, T. (2006). The igraph software package for complex network research. InterJournal Complex Systems, 1695, 5133–9.

[ece35071-bib-0018] DeMars, C. A. , Auger‐Méthé, M. , Schlägel, U. E. , & Boutin, S. (2013). Inferring parturition and neonate survival from movement patterns of female ungulates: A case study using woodland caribou. Ecology and Evolution, 3, 4149–4160. 10.1002/ece3.785 24324866 PMC3853560

[ece35071-bib-0019] Doligez, B. , Danchin, E. , & Clobert, J. (2002). Public information and breeding habitat selection in a wild bird population. Science, 297, 1168–1171. 10.1126/science.1072838 12183627

[ece35071-bib-0020] Environment and Climate Change Canada . (2017). Historical climate data. http://climate.weather.gc.ca/

[ece35071-bib-0021] Faille, G. , Dussault, C. , Ouellet, J.‐P. , Fortin, D. , Courtois, R. , St‐Laurent, M.‐H. , & Dussault, C. (2010). Range fidelity: The missing link between caribou decline and habitat alteration? Biological Conservation, 143, 2840–2850.

[ece35071-bib-0022] Farine, D. R. (2014). Measuring phenotypic assortment in animal social networks: Weighted associations are more robust than binary edges. Animal Behaviour, 89, 141–153. 10.1016/j.anbehav.2014.01.001

[ece35071-bib-0023] Farine, D. R. (2015). Proximity as a proxy for interactions: Issues of scale in social network analysis. Animal Behaviour, 104, e1–e5.

[ece35071-bib-0024] Farine, D. R. , & Whitehead, H. (2015). Constructing, conducting and interpreting animal social network analysis. Journal of Animal Ecology, 84, 1144–1163. 10.1111/1365-2656.12418 26172345 PMC4973823

[ece35071-bib-0025] Fieberg, J. , & Kochanny, C. O. (2005). Quanitfying home‐range overlap: The importance of the utilization distribution. Journal of Wildlife Management, 69, 1346–1359.

[ece35071-bib-0026] Fletcher, R. J. (2006). Emergent properties of conspecific attraction in fragmented landscapes. The American Naturalist, 168, 207–219. 10.1086/505764 16874630

[ece35071-bib-0027] Fortin, D. , & Fortin, M. E. (2009). Group‐size‐dependent association between food profitability, predation risk and distribution of free‐ranging bison. Animal Behaviour, 78, 887–892. 10.1016/j.anbehav.2009.06.026

[ece35071-bib-0028] Gero, S. , Gordon, J. , & Whitehead, H. (2013). Calves as social hubs: Dynamics of the social network within sperm whale units. Proceedings of the Royal Society B, 280, 20131113 10.1098/rspb.2013.1113 23740785 PMC3774244

[ece35071-bib-0029] Hansen, B. B. , Aanes, R. , & Sæther, B. (2010). Feeding‐crater selection by high‐arctic reindeer facing ice‐blocked pastures. Canadian Jounal of Zoology, 88, 170–177. 10.1139/Z09-130

[ece35071-bib-0030] Johnson, D. D. P. , Kays, R. , Blackwell, P. G. , & Macdonald, D. W. (2002). Does the resource hypothesis explain group living? Trends in Ecology & Evolution, 17, 563–570.

[ece35071-bib-0031] Johnson, D. D. P. , & Macdonald, D. W. (2003). Sentenced without trial: Reviling and revamping the resource dispersion hypothesis. Oikos, 101, 433–440. 10.1034/j.1600-0706.2003.12697.x

[ece35071-bib-0032] Kawaguchi, L. G. , Ohashi, K. , & Toquenaga, Y. (2006). Do bumble bees save time when choosing novel flowers by following conspecifics? Functional Ecology, 20, 239–244.

[ece35071-bib-0033] Lafontaine, A. , Drapeau, P. , Fortin, D. , & St‐Laurent, M.‐H. (2017). Many places called home: The adaptive value of seasonal adjustments in range fidelity. Journal of Animal Ecology, 86, 624–633. 10.1111/1365-2656.12645 28146328

[ece35071-bib-0034] Lesmerises, F. , Johnson, C. J. , & St‐Laurent, M.‐H. (2018). Landscape knowledge is an important driver of the fission dynamics of an alpine ungulate. Animal Behaviour, 140, 39–47. 10.1016/j.anbehav.2018.03.014

[ece35071-bib-0035] Lewis, K. P. , & Mahoney, S. P. (2014). Caribou survival, fate, and cause of mortality in Newfoundland: A summary and analysis of the patterns and causes of caribou survival and mortality in Newfoundland during a period of rapid population decline (2003–2012). Technical Bulletin No. 009, Sustainable Development and Strategic Science. St. John's, NL: Government of Newfoundland and Labrador.

[ece35071-bib-0036] Leyrer, J. , Spaans, B. , Camara, M. , & Piersma, T. (2006). Small home ranges and high site fidelity in red knots (*Calidris c. canutus*) wintering on the Banc d'Arguin. Mauritania. Journal of Ornithology, 147, 376–384. 10.1007/s10336-005-0030-8

[ece35071-bib-0037] Lingle, S. (2001). Anti‐predator strategies and grouping patterns in white tailed deer and mule deer. Ethology, 107, 295–314. 10.1046/j.1439-0310.2001.00664.x

[ece35071-bib-0038] Lovell, P. G. , Ruxton, G. D. , Langridge, K. V. , & Spencer, K. A. (2013). Egg‐laying substrate selection for optimal camouflage by quail. Current Biology, 23, 260–264. 10.1016/j.cub.2012.12.031 23333313

[ece35071-bib-0039] MacDonald, D. W. (1983). The ecology of carnivore social behaviour. Nature, 301, 379–384. 10.1038/301379a0

[ece35071-bib-0040] MacDonald, D. W. , & Johnson, D. D. P. (2015). Patchwork planet: The resource dispersion hypothesis, society, and the ecology of life. Journal of Zoology, 295, 75–107. 10.1111/jzo.12202

[ece35071-bib-0041] MacNearney, D. , Pigeon, K. , Stenhouse, G. , Nijland, W. , Coops, N. , & Finnegan, L. (2016). Heading for the hills? Evaluating spatial distribution of woodland caribou in response to a growing anthropogenic disturbance footprint. Ecology and Evolution, 6, 6484–6509. 10.1002/ece3.2362 27777724 PMC5058522

[ece35071-bib-0042] Mahoney, S. P. , & Virgl, J. A. (2003). Habitat selection and demography of a nonmigratory woodland caribou population in Newfoundland. Canadian Journal of Zoology, 81, 321–334. 10.1139/z02-239

[ece35071-bib-0043] Makin, D. F. , Chamaillé‐Jammes, S. , & Shrader, A. M. (2017). Herbivores employ a suite of antipredator behaviours to minimize risk from ambush and cursorial predators. Animal Behaviour, 127, 225–231. 10.1016/j.anbehav.2017.03.024

[ece35071-bib-0044] Mallory, C. D. , & Boyce, M. S. (2017). Observed and predicted effects of climate change on Arctic caribou and reindeer. Environmental Review, 13, 5133–5145.

[ece35071-bib-0045] Mayor, S. J. , Schaefer, J. A. , Schneider, D. C. , & Mahoney, S. P. (2009). The spatial structure of habitat selection: A caribou's‐eye‐view. Acta Oecologica, 35, 253–260. 10.1016/j.actao.2008.11.004

[ece35071-bib-0046] Mcloughlin, P. D. , Ferguson, S. H. , & Messier, F. (2000). Intraspecific variation in home range overlap with habitat quality: A comparison among brown bear populations. Evolutionary Ecology, 14, 39–60. 10.1023/A:1011019031766

[ece35071-bib-0047] Merkle, J. A. , Sigaud, M. , & Fortin, D. (2015). To follow or not? How animals in fusion‐fission societies handle conflicting information during group decision‐making. Ecology Letters, 18, 799–806. 10.1111/ele.12457 26013202

[ece35071-bib-0048] Moll, R. J. , Redilla, K. M. , Mudumba, T. , Muneza, A. B. , Gray, S. M. , Abade, L. , … Montgomery, R. A. (2017). The many faces of fear: A synthesis of the methodological variation in characterizing predation risk. Journal of Animal Ecology, 86, 749–765. 10.1111/1365-2656.12680 28390066

[ece35071-bib-0049] Morse, D. H. (1999). Choice of hunting site as a consequence of experience in late‐instar crab spiders. Oecologia, 120, 252–257. 10.1007/s004420050855 28308086

[ece35071-bib-0050] Orrock, J. L. , Grabowski, J. H. , Pantel, J. H. , Peacor, S. D. , Peckarsky, B. L. , Sih, A. , & Werner, E. E. (2008). Consumptive and nonconsumptive effects of predators on metacommunities of competing prey. Ecology, 89, 2426–2435. 10.1890/07-1024.1 18831164

[ece35071-bib-0051] Pruitt, W. O. Jr (1959). Snow as a factor in the winter ecology of the barren ground caribou (*Rangifer arcticus*). Arctic, 12, 158–179.

[ece35071-bib-0052] R Core Team . (2017). R: A language and environment for statistical computing. Vienna, Austria: R Foundation for Statistical Computing.

[ece35071-bib-0053] Ray, C. , Gilpin, M. , & Smith, A. T. (1991). The effect of conspecific attraction on metapopulation dynamics. Biological Journal of the Linnean Society, 42, 123–134. 10.1111/j.1095-8312.1991.tb00555.x

[ece35071-bib-0054] Revilla, E. (2003). What does the resource dispersion hypothesis explain, if anything? Oikos, 101, 428–432.

[ece35071-bib-0055] Robertson, A. , Palphramand, K. L. , Carter, S. P. , & Delahay, R. J. (2015). Group size correlates with territory size in European badgers: Implications for the resource dispersion hypothesis? Oikos, 124, 507–514.

[ece35071-bib-0056] Robitaille, A. L. , Webber, Q. M. R. , & Vander, E. (2018). Conducting social network analysis with animal telemetry data: applications and methods using spatsoc. bioarXiv, 447284 10.1101/447284

[ece35071-bib-0057] Rominger, E. M. , Robbins, C. T. , & Evans, M. A. (1996). Winter foraging ecology of woodland Caribou in Northeastern Washington. The Journal of Wildlife Management, 60, 719–728. 10.2307/3802370

[ece35071-bib-0058] Schaefer, J. A. , Bergman, C. M. , & Luttich, S. N. (2000). Site fidelity of female caribou at multiple spatial scales. Landscape Ecology, 15, 731–739.

[ece35071-bib-0059] Schaefer, J. A. , & Mahoney, S. P. (2007). Effects of progressive Clearcut logging on Newfoundland Caribou. The Journal of Wildlife Management, 71, 1753–1757. 10.2193/2005-479

[ece35071-bib-0060] Schaefer, J. A. , & Mahoney, S. P. (2013). Spatial dynamics of the rise and fall of caribou (*Rangifer tarandus*) in Newfoundland. Canadian Journal of Zoology, 91, 767–774.

[ece35071-bib-0061] Schuck‐Paim, C. , & Alonso, W. J. (2001). Deciding where to settle: Conspecific attraction and web site selection in the orb‐web spider *Nephilengys cruentata* . Animal Behaviour, 61, 1007–1012. 10.1006/anbe.2001.1841

[ece35071-bib-0062] Sikes, R. S. , & Gannon, W. L. (2011). Guidelines of the American Society of Mammalogists for the use of wild mammals in research. Journal of Mammalogy, 92, 235–253. 10.1644/10-MAMM-F-355.1 PMC5909806 29692469

[ece35071-bib-0063] Spiegel, O. , Leu, S. T. , Sih, A. , & Bull, C. M. (2016). Socially interacting or indifferent neighbours? Randomization of movement paths to tease apart social preference and spatial constraints. Methods in Ecology and Evolution, 7, 971–979. 10.1111/2041-210X.12553

[ece35071-bib-0064] Stamps, J. A. (1988). Conspecific attraction and aggregation in territorial species. The American Naturalist, 131, 329–347. 10.1086/284793

[ece35071-bib-0065] Stuart‐Smith, A. K. , Bradshaw, C. J. A. , Boutin, S. , Hebert, D. M. , & Rippin, A. B. (1997). Woodland caribou relative to landscape patterns in northeastern Alberta. The Journal of Wildlife Management, 61, 622–633. 10.2307/3802170

[ece35071-bib-0066] Switzer, P. V. (1993). Site fidelity in predictable and unpredictable habitats. Evolutionary Ecology, 7, 533–555. 10.1007/BF01237820

[ece35071-bib-0067] van Beest, F. M. , Vander Wal, E. , Stronen, A. V. , Paquet, P. C. , & Brook, R. K. (2013). Temporal variation in site fidelity: Scale‐dependent effects of forage abundance and predation risk in a non‐migratory large herbivore. Oecologia, 173, 409–420. 10.1007/s00442-013-2647-2 23552985

[ece35071-bib-0068] Van Moorter, B. , Rolandsen, C. M. , Basille, M. , & Gaillard, J.‐M. (2016). Movement is the glue connecting home ranges and habitat selection. Journal of Animal Ecology, 85, 21–31. 10.1111/1365-2656.12394 25980987

[ece35071-bib-0069] Van Moorter, B. , Visscher, D. , Benhamou, S. , Börger, L. , Boyce, M. S. , & Gaillard, J.‐M. (2009). Memory keeps you at home: A mechanistic model for home range emergence. Oikos, 118, 641–652. 10.1111/j.1600-0706.2008.17003.x

[ece35071-bib-0070] Vors, L. S. , & Boyce, M. S. (2009). Global declines of caribou and reindeer. Global Change Biology, 15, 2626–2633. 10.1111/j.1365-2486.2009.01974.x

[ece35071-bib-0071] Webber, Q. M. R. , & Vander Wal, E. (2018). An evolutionary framework outlining the integration of individual social and spatial ecology. Journal of Animal Ecology, 87, 113–127. 10.1111/1365-2656.12773 29055050

[ece35071-bib-0072] Whitehead, H. (2008). Analyzing animal societies: Quantitative methods for vertebrate social analysis. Chicago, IL: University of Chicago Press.

[ece35071-bib-0073] Wittmer, H. U. , McLellan, B. N. , & Hovey, F. W. (2006). Factors influencing variation in site fidelity of woodland caribou (*Rangifer tarandus caribou*) in southeastern British Columbia. Canadian Journal of Zoology, 84, 537–545.

[ece35071-bib-0074] Wolf, J. B. W. , & Trillmich, F. (2007). Beyond habitat requirements: Individual fine‐scale site fidelity in a colony of the Galapagos sea lion (*Zalophus wollebaeki*) creates conditions for social structuring. Oecologia, 152, 553–567. 10.1007/s00442-007-0665-7 17505851

[ece35071-bib-0075] Worton, B. J. (1989). Kernel methods for estimating the utilization distribution in home‐range studies. Ecology, 70, 164–168. 10.2307/1938423

